# Comparison of Healthcare Utilization Before Diagnosis Between Non‐Advanced and Advanced Lung Cancer Patients: A Study Among 72,851 Cases in Germany

**DOI:** 10.1002/ijc.70682

**Published:** 2026-08-27

**Authors:** Josephine Küsel‐Kanbach, Oliver Riedel, Ulrike Haug

**Affiliations:** ^1^ Department of Clinical Epidemiology Leibniz Institute for Prevention Research and Epidemiology – BIPS Bremen Germany; ^2^ Faculty of Human and Health Sciences University of Bremen Bremen Germany

**Keywords:** comorbidity, lung cancer, stage, utilization of healthcare system

## Abstract

There is no study from Germany on the association between stage of lung cancer (LC) and patient characteristics. We aimed to compare advanced versus non‐advanced LC cases regarding indicators of healthcare utilization. Using claims data covering 20% of the German population, we included LC patients diagnosed between 2013 and 2019. Stratified by stage (advanced vs. non‐advanced), we assessed comorbidities, hospitalizations, outpatient physician contacts and utilization of preventive measures before LC diagnosis. Among 72,851 included LC patients, 29% (females) and 28% (males) were classified as non‐advanced. For all comorbidities considered and for most other indicators, an inverse association with stage was observed. For example, in males with non‐advanced versus advanced LC, the proportions with chronic obstructive pulmonary disease were 37% and 27% (females: 38% vs. 29%), and the proportions with ≥ 1 hospitalization in the 3 years prior LC diagnosis were 70% versus 58% (females: 69% vs. 55%). In conclusion, LC patients with more frequent healthcare utilization before LC diagnosis were more likely to be diagnosed at a non‐advanced stage. Regarding future LC screening, the findings underline the importance of developing implementation strategies that ensure the reach of those who use health services less frequently, as these individuals are at higher risk of being diagnosed at an advanced stage if they develop LC.

## Abstract (highlights)

What's new?

The surveillance hypothesis states that patients with comorbidities have more frequent contacts with the healthcare system and therefore a higher chance to be diagnosed early for lung cancer. Using claims data covering 20% of the German population, this study found a strong association between indicators of healthcare utilization and stage at lung cancer diagnosis in Germany. Patients treated for comorbidities or hospitalized within 3 years before lung cancer diagnosis were significantly more often detected at an early stage. The findings underline the importance of developing strategies for lung cancer screening that also reach patients who use health services less frequently.

## Graphical abstract

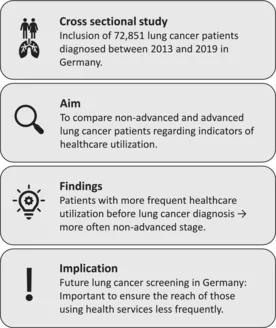

**Abbreviations**
AOK: Allgemeine Ortskrankenkasse
COPD: Chronic obstructive pulmonary disease
FOBT: Fecal occult blood test
GePaRD: German Pharmacoepidemiological Research Database
ICD‐10 GM: International Statistical Classification of Diseases and Related Health Problems, 10th revision, German Modification
LC: Lung cancer
UICC: Union internationale contre le cancer

## 1 Introduction

In Germany, lung cancer (LC) is the most common cause of cancer death in men and the second most common in women. For the years 2021–2023, the five‐year relative survival rates were 19% among men and 25% among women. In the same years, 76% of female and 78% of male LC patients were diagnosed at UICC (Union Internationale Contre le Cancer) Stage III and IV according to cancer registry data [1]. LC mortality could be lowered if more LC cases were diagnosed at a non‐advanced stage. However, symptoms of LC rarely occur in non‐advanced stages and are unspecific, such as cough and chest pain [2]. In a nationwide multicentre study in Spain including 9876 LC patients, Ruano‐Raviña et al. found that 59% of non‐small cell LC patients with UICC Stage I and 42% of those with UICC Stage II did not have any symptoms [2].

Previous studies from other countries reported a higher chance of being diagnosed at a non‐advanced stage in LC patients with comorbidities [3, 4, 5, 6, 7, 8, 9]. This observation has been discussed as the surveillance hypothesis, stating that patients with comorbidities have more frequent contacts to the healthcare system and therefore a higher chance to be diagnosed at an early cancer stage [10, 11]. However, no study investigating such associations has been conducted in Germany.

Therefore, this paper aims to characterize and compare LC patients diagnosed at a non‐advanced versus advanced stage with respect to comorbidities prior to the LC diagnosis and other factors reflecting the extent to which the healthcare system was utilized. We used health claims data because—unlike cancer registry data—they also contain information on comorbidity and utilization of the healthcare system.

## 2 Materials and Methods

### 2.1 Data Source

We used the so‐called German Pharmacoepidemiological Research Database (GePaRD), which is based on claims data from four statutory health insurance providers in Germany and currently includes information on approximately 25 million persons who have been insured with one of the participating providers since 2004 or later. Besides, we also used data from another statutory health insurance provider (AOK Nordwest; 1,518,267 persons). In addition to demographic data, health claims data contain information on drug dispensations as well as outpatient (i.e., from general practitioners and specialists) and inpatient services and diagnoses. Per data year, there is information on approximately 20% of the general population and all geographical regions of Germany are represented. In Germany, membership in statutory health insurance is mandatory except, for example, for persons with a very high income or civil servants. Overall, about 87% of the population are insured through statutory health insurance providers. In the German statutory health insurance system, there is a uniform access to all levels of care [12].

To identify incident LC patients (International statistical classification of diseases, 10th Revision German Modification (ICD‐10 GM): C33‐C34), we used a previously developed algorithm showing plausible results in regard to LC incidence and stage distribution when compared to cancer registry data [13]. UICC stage is not available in German claims data, but we categorized LC patients into advanced versus non‐advanced stage at diagnosis based on ICD codes indicating lymph node involvement or distant metastases as well as ICD codes indicating locally advanced stage (ICD‐10 GM: C34.8) [14].

### 2.2 Study Population and Study Design

We conducted a cross‐sectional study including incident LC patients diagnosed between 2013 and 2019 with at least 3 years of continuous pre‐observation period (gaps of 30 days allowed). We excluded patients with missing or inconsistent information on sex or birth year and patients not residing in Germany. Included persons were classified into non‐advanced and advanced stage at LC diagnosis. These two groups were characterized regarding comorbidity and healthcare utilization as well as indicators of the socioeconomic status.

### 2.3 Characterization of Patients With Non‐Advanced Versus Advanced LC

Regarding indicators of the socioeconomic status, we classified patients based on the district of residence into high, medium, and low deprivation index using the German Index of Socioeconomic Deprivation [15].

We determined the number of outpatient contacts, hospitalizations as well as the number of days spent in the hospital based on the pre‐observation period to capture healthcare utilization in the 3 years prior to LC diagnosis. Furthermore, we assessed utilization of preventive measures reimbursed by statutory health insurances during this period. This includes a general health check‐up as well as mammography screening, fecal occult blood testing (FOBT), skin cancer screening, cervical cancer screening and prostate cancer screening (all offered at intervals ≤ 3 years in the age range relevant for this study). Considering the patients' full database history (i.e., not only the 3 years before the LC diagnosis) we assessed participation in screening colonoscopy (offered twice at a 10‐year interval) and screening for abdominal aortic aneurysms (offered only once) since, given the nature of these screening offers, a three‐year period would be somewhat short.

To characterize patients regarding comorbidities, we selected those that were often considered in prior studies in the field, which had a particular focus on comorbidities underlying the Charlson Comorbidity Index. To define these comorbidities in health claims data, we used previously developed algorithms that take into account potential miscoding—particularly in the outpatient setting—and thus ensure a high specificity [16]. We assessed these comorbidities based on the patients' full database history. Since the selected comorbidities are chronic conditions that generally require ongoing treatment, we do not expect—also based on insights from previous analyses—that the prevalence would have changed significantly if the assessment period had been limited to 3 years. Further, we characterized patients regarding indicators of an unhealthy lifestyle including codes for alcohol abuse, smoking, and obesity. Here, we used the patients' entire database history to increase sensitivity, especially since these conditions may not be coded on a regular basis but are usually persistent.

### 2.4 Data Analysis

We conducted descriptive analyses to characterize and compare LC patients with non‐advanced versus advanced stage at diagnosis regarding the variables mentioned above using counts, proportions, means, and standard deviations where appropriate. For proportions, we calculated Wilson 95% confidence intervals.

We performed two sensitivity analyses. First, we also classified those patients as advanced who died within the same quarter or in the quarter after the LC diagnosis, irrespective of other codes. In the main analyses, these patients were only classified as advanced if there were codes indicating advanced stage as described above; otherwise, they were classified as non‐advanced in the main analysis. Second, we stratified all analyses by age group (< 55 years, 55–64 years, 65–74 years, and ≥ 75 years) to assess potential differences independent of age. We also calculated odds ratios adjusted for age.

All statistical analyses were conducted using SAS 9.4.

## 3 Results

### 3.1 Sociodemographic Characteristics

We included 30,862 female and 41,989 male LC patients, of which 29% and 28% were classified as non‐advanced, respectively (Table S1). Patients with non‐advanced LC were on average 2 years older compared to advanced LC patients. Both in male and female patients, the distribution of the deprivation index of the district of residence was similar in non‐advanced and advanced stages.

### 3.2 Healthcare Utilization

In females, 93% of non‐advanced versus 94% of advanced LC patients had at least one contact to a physician in the outpatient setting 3 years prior to the LC diagnosis. Among men, these proportions were 90% versus 92%, respectively. Both in males and females, the mean number of outpatient contacts among those with at least one outpatient visit was 16% higher in non‐advanced versus advanced LC cases. Among females, 69% of non‐advanced versus 55% of advanced LC patients (males: 70% vs. 58%) were at least once hospitalized in the 3 years prior to the LC diagnosis (Table 1).

**TABLE 1:** Outpatient contacts and hospitalizations in the 3 years before LC diagnosis, stratified by stage at diagnosis (advanced vs. non‐advanced) and sex.

	Women			Men		
	Non‐advanced *n* = 8842	Advanced *n* = 22,020	Relative difference^a^	Non‐advanced *n* = 11,722	Advanced *n* = 30,267	Relative difference^a^
Outpatient contacts						
At least one outpatient contact, *n* (%)	8221 (93.0)	20,708 (94.0)	0.99	10,596 (90.4)	27,883 (92.1)	0.98
95% CI	92.4–93.5	93.7–94.3		89.8–90.9	91.8–92.4	
Among patients with at least one outpatient contact						
Mean (SD)	39.7 (21.9)	34.3 (20.3)	1.16	35.6 (21.0)	30.6 (19.7)	1.16
Median (IQR)	36.0 (24.0–52.0)	31.0 (20.0–45.0)	1.16	33.0 (20.0–47.0)	28.0 (16.0–42.0)	1.18
Hospitalizations						
At least one hospitalization, *n* (%)	6127 (69.3)	12,127 (55.1)	1.26	8197 (69.9)	17,512 (57.9)	1.21
95% CI	68.3–70.2	54.4–55.7		69.1–70.8	57.3–58.4	
Among patients with at least one hospitalization						
Mean (SD)	3.0 (2.7)	2.6 (2.5)	1.15	3.2 (2.9)	2.8 (2.7)	1.14
Median (IQR)	2.0 (1.0–4.0)	2.0 (1.0–3.0)	1.00	2.0 (1.0–4.0)	2.0 (1.0–3.0)	1.00
Duration of hospital stays in days						
Mean (SD)	28.9 (52.1)	24.5 (43.7)	1.19	29.0 (47.2)	24.5 (41.4)	1.18
Median (IQR)	15.0 (6.0–33.0)	12.0 (5.0–28.0)	1.25	15.0 (7.0–35.0)	12.0 (5.0–28.0)	1.25

Abbreviations: CI, Confidence interval; IQR, Range from the lower to the upper quartile; SD, Standard deviation.

^a^ The relative difference is calculated as the proportion, median or mean among non‐advanced LC patients divided by the proportion, median or mean among advanced LC patients, respectively.

The relative difference in the proportion participating in preventive measures in the 3 years prior to the LC diagnosis was mostly below 8%. Only for mammography screening and colonoscopy screening among women, the relative difference was about 10% (Table 2).

**TABLE 2:** Participation in preventive measures before LC diagnosis, stratified by stage at diagnosis (advanced vs. non‐advanced) and sex.

	Women			Men		
	Non‐advanced *n* = 8842	Advanced *n* = 22,020	Relative difference^e^	Non‐advanced *n* = 11,722	Advanced *n* = 30,267	Relative difference^e^
	% (95% CI)	% (95% CI)	Ratio	% (95% CI)	% (95% CI)	Ratio
General health check‐up (“Check‐up 35”)^a^	60.8 (59.8–61.8)	60.5 (59.8–61.1)	1.00	57.4 (56.5–58.3)	57.7 (57.1–58.2)	0.99
Mammography screening^a^	24.2 (23.3–25.1)	26.9 (26.3–27.5)	0.90	—	—	—
Gynecological examination^a^, ^c^	41.6 (40.6–42.7)	41.6 (41.0–42.3)	1.00	—	—	—
Urological examination^a^, ^d^	—	—	—	39.1 (38.3–40.0)	36.7 (36.2–37.3)	1.07
Skin cancer screening^a^	38.3 (37.3–39.3)	37.8 (37.1–38.4)	1.01	37.0 (36.1–37.9)	36.4 (35.9–37.0)	1.02
Colonoscopy screening^b^	15.4 (14.7–16.2)	13.9 (13.5–14.4)	1.11	14.9 (14.3–15.6)	13.8 (13.4–14.2)	1.08
Fecal Occult Blood Test (FOBT)^a^	20.5 (19.7–21.4)	21.1 (20.6–21.6)	0.98	18.5 (17.8–19.2)	18.3 (17.9–18.7)	1.01
Screening for abdominal aortic aneurysm^b^	—	—	—	2.8 (2.6–3.2)	2.7 (2.6–2.9)	1.04

Abbreviation: CI, Confidence interval.

^a^ 3 years prior to LC diagnosis.

^b^ Ever before the LC diagnosis.

^c^ The reimbursement code “gynecological examination” includes early detection of cervical cancer by cytology and breast palpation.

^d^ This reimbursement code includes digital rectal examination of the prostate, examination of external genitalia, and query of medical history.

^e^ The relative difference is calculated as the proportion among non‐advanced LC patients divided by the proportion among advanced LC patients.

### 3.3 Comorbidities and Other Conditions

For all of the comorbidities considered, the proportion of LC patients with the respective comorbidity was higher in non‐advanced versus advanced cases, both for women and men (Table 3). Among women, the highest relative difference was observed for the treatment with anticoagulant therapies (relative difference: 51%), followed by treated bronchial asthma (relative difference: 46%), congestive heart failure (relative difference: 46%), hemiplegia (relative difference: 38%), treatment with immunosuppressive drugs (relative difference: 37%), coronary heart disease (relative difference: 33%), and COPD (relative difference: 31%). Among men, the highest relative difference was observed for treated bronchial asthma (relative difference: 55%), treatment with immunosuppressive drugs (relative difference: 44%), COPD (relative difference: 37%), congestive heart failure (relative difference: 37%), treatment with anticoagulant therapies (relative difference: 26%), treatment with platelet aggregation inhibitors (relative difference: 20%), and coronary heart disease (relative difference: 20%). The proportion with at least two of the considered comorbidities was 33% (women) and 21% (men) higher among non‐advanced LC patients as compared to advanced LC patients. For indicators of an unhealthy lifestyle, the proportions with the respective codes also tended to be higher among non‐advanced cases, but the relative differences were lower as compared to comorbidities (women: all ≤ 11%; men: all ≤ 5%). For most of the differences, the 95% confidence intervals of the proportions for non‐advanced and advanced patients were non‐overlapping.

**TABLE 3:** Comorbidities of LC patients present before LC diagnosis, stratified by stage at diagnosis (advanced vs. non‐advanced) and sex.

	Women					Men				
	Non‐advanced *n* = 8842		Advanced *n* = 22,020		Relative difference^b^	Non‐advanced *n* = 11,722		Advanced *n* = 30,267		Relative difference^b^
	%	95% CI	%	95% CI	Ratio	%	95% CI	%	95% CI	Ratio
Comorbidities										
Coronary heart disease	21.3	20.4–22.1	16.0	15.6–16.5	1.33	34.3	33.4–35.1	28.6	28.1–29.1	1.20
Congestive heart failure	13.1	12.4–13.8	9.0	8.7–9.4	1.46	16.7	16.1–17.4	12.2	11.8–12.6	1.37
Myocardial infarction	3.7	3.3–4.1	2.9	2.7–3.1	1.28	7.4	6.9–7.9	6.2	5.9–6.4	1.19
Stroke	5.0	4.6–5.5	3.9	3.6–4.2	1.28	6.6	6.2–7.0	5.8	5.6–6.1	1.14
Platelet aggregation inhibitors	7.0	6.5–7.6	5.6	5.3–5.9	1.25	13.0	12.4–13.6	10.8	10.4–11.1	1.20
Treatment with acetylsalicylic acid	14.8	14.0–15.5	11.4	11.0–11.9	1.30	23.1	22.4–23.9	20.0	19.5–20.4	1.16
Dual antiplatelet therapy	3.5	3.1–3.9	2.8	2.6–3.0	1.25	6.9	6.4–7.3	5.9	5.6–6.1	1.17
Treatment with anticoagulant therapy	10.6	10.0–11.3	7.0	6.7–7.4	1.51	14.9	14.3–15.6	11.8	11.5–12.2	1.26
Treatment with lipid‐modifying agents	29.1	28.2–30.1	25.4	24.8–26.0	1.15	36.5	35.6–37.4	33.3	32.7–33.8	1.10
Drug‐treated hypertension	59.1	58.0–60.1	53.2	52.5–53.8	1.11	61.3	60.4–62.2	56.4	55.9–57.0	1.09
COPD	38.0	37.0–39.0	29.1	28.5–29.7	1.31	36.9	36.0–37.8	26.9	26.4–27.4	1.37
Treated bronchial asthma	13.4	12.8–14.2	9.2	8.8–9.5	1.46	8.5	8.0–9.0	5.5	5.2–5.7	1.55
Liver diseases including chronic viral hepatitis	17.3	16.6–18.1	15.9	15.5–16.4	1.09	20.0	19.3–20.8	19.2	18.8–19.7	1.04
Treated diabetes	12.7	12.0–13.4	11.1	10.7–11.6	1.14	18.6	17.9–19.3	17.6	17.1–18.0	1.06
Treatment with immunosuppressive drugs	14.7	14.0–15.4	10.7	10.3–11.1	1.37	12.1	11.5–12.7	8.4	8.1–8.7	1.44
Hemiplegia	5.1	4.7–5.6	3.7	3.5–4.0	1.38	5.6	5.2–6.1	4.9	4.6–5.1	1.14
At least two of the above^c^	46.5	45.5–47.6	35.0	34.4–35.7	1.33	54.8	53.9–55.7	45.2	44.6–45.7	1.21
Indicators of unhealthy lifestyle										
Alcohol abuse	10.4	9.8–11.0	10.0	9.6–10.4	1.04	18.3	17.6–19.0	17.5	17.0–17.9	1.05
Smoking^a^	44.3	43.2–45.3	45.1	44.5–45.8	0.98	45.4	44.5–46.3	44.6	44.1–45.2	1.02
Obesity	29.2	28.3–30.2	26.4	25.8–27.0	1.11	26.5	25.7–27.3	26.2	25.8–26.7	1.01

Abbreviations: CI, Confidence interval; COPD, Chronic obstructive pulmonary disease.

^a^ Information on smoking is obtained from the claims data via information on mental and behavioral disorders due to use of tobacco (ICD‐10 GM: F17), tobacco or nicotine poisoning (T65.2), or by prescribing medication to treat nicotine dependence (ATC‐Code: N07BA). As information on smoking is not routinely collected, the proportion of smokers is likely underestimated in German health claims data.

^b^ The relative difference is calculated as the proportion among non‐advanced LC patients divided by the proportion among advanced LC patients.

^c^ Less severe comorbidities (treatment with lipid‐modifying agents and drug‐treated hypertension) were not considered here.

The sensitivity analysis regarding stage classification showed similar results as the main analyses (Tables S2–S5). The stratification by age group showed similar results compared to the main analyses for outpatient contacts and hospitalizations (Table S6) and comorbidities (Table S8). Regarding the uptake of preventive measures, age stratification partly increased differences between groups in the direction as expected (higher uptake among non‐advanced cases (Table S7)). The impact of age on the comparison of advanced versus non‐advanced cases is also reflected by the comparison of unadjusted and age‐adjusted odds ratios shown in Tables S9 and S10.

## 4 Discussion

Our study including 72,851 patients with LC provides several indicators that patients diagnosed at an early stage utilized the healthcare system in the 3 years before diagnosis more than those diagnosed at an advanced stage. Our study also suggests that the intensity of received healthcare matters: The association was particularly pronounced when considering treated comorbidities or hospitalizations as proxies for healthcare utilization. The associations with the uptake of preventive measures were weaker but still in the same direction. The observed patterns were consistent in men and women.

To the best of our knowledge, there is no other study from Germany comparing non‐advanced versus advanced stage LC patients regarding comorbidity or the utilization of preventive healthcare. Internationally, there are a few studies comparing non‐advanced and advanced stage LC patients regarding comorbidity, but mainly using a comorbidity index only [4, 5, 6, 7, 8, 17, 18] or selected single comorbidities such as COPD [17, 19, 20]. None of these studies reported on the association between utilization of preventive measures and stage at diagnosis. Although this limits comparability, it should be noted that the associations can vary from country to country anyway due to differences between healthcare systems. Nonetheless, the existing studies, which were conducted in the United States, Canada, the United Kingdom, Denmark, and the Netherlands, all reported an inverse association between comorbidity and stage at diagnosis, that is, similar to our study; only in two studies, positive associations were observed for single comorbidities such as congestive heart failure, but for other comorbidities such as COPD and a comorbidity index, they consistently found an inverse association [3, 9, 19].

Thus, our findings and the aforementioned studies support the hypothesis that more frequent contacts to the healthcare system due to comorbidity increase the chance of LC to be detected at an earlier stage. The underlying mechanisms may include the incidental detection of a preclinical stage in the context of examining other conditions (e.g., imaging) or detection of a clinical stage with unspecific symptoms noticed by physicians and then followed up. Regardless of comorbidity, another hypothesis could be that individuals who generally use more preventive health services are also more likely to see a physician for unspecific symptoms leading to earlier LC detection. Although our data also partially support this hypothesis, the fact that the association between non‐advanced stage LC and comorbidities was much stronger as compared to the association with the uptake of preventive measures suggests that comorbidity‐related healthcare contacts play a particularly important role as an underlying mechanism. It is plausible that screening‐related healthcare contacts are rather focused on the respective examinations, that is, there is less of a chance of detecting other diseases. On the other hand, the lack of a (relevant) association with the general health check‐up was somewhat surprising (only in men was a weak association observed after age adjustment, see Table S9), as this examination should actually have a broader focus.

Regarding comorbidities, LC patients can be categorized into two groups: patients with comorbidities according to claims data (i.e., recorded in claims data) and those without such comorbidities. The group without comorbidities according to claims data is likely to comprise two subgroups: one part may still have these conditions but does not seek medical care for them. In a recently conducted study on mammography screening in Germany, for example, there was a subgroup of women who had a low prevalence of treated hypertension at baseline but died from cardiovascular disease more often than other women [21]. Another part may actually be free of these comorbidities. Distinguishing these groups is important when discussing the relevance of our findings with respect to future LC screening based on low‐dose computer tomography in Germany, which has been offered to (former) heavy smokers aged 50–75 years since April 2026 [22]. The subgroup of patients (actually) free of comorbidities would likely have the highest benefit from early LC detection as they are less likely to die from diseases other than LC. This highlights the need to organize future LC screenings in such a way that the most relevant target groups are reached.

In the interpretation of our results, it should be considered that this study was designed as a first step to assess whether an association between healthcare utilization and stage at LC diagnosis exists at all, but it does not provide information on potential mechanisms underlying the surveillance hypothesis. Exploring these mechanisms including patient journeys is important and therefore represents a logical next step that should be addressed in future studies. Besides, it should be noted that they focus on LC and may not be generalizable, that is, further studies are needed investigating the association between stage at diagnosis and healthcare utilization for other cancers. A meta‐analysis of studies on lung, colorectal, breast, and prostate cancer suggested that there are some differences between cancers [11], but these studies were all conducted in other countries except for one study on breast cancer from 2001 [23]. Besides, our results should not be transferred to the association between LC stage and comorbidities not considered in our study as the impact might be different. As a limitation, it should be considered that only a rough stage classification is possible with health claims data. Further, it mainly relies on ICD codes for lymph node involvement and distant metastases, which may not be coded if the cancer is too advanced to be treated. Reassuringly, however, the results of sensitivity analyses, classifying patients who died in the same or the subsequent quarter of LC diagnosis as advanced irrespective of ICD codes used in the main analyses, confirmed the results of the main analyses. In other words, the sensitivity analyses confirmed that our conclusion is robust regarding potential misclassification in this regard. Therefore, we do not believe that the fact that we used health claims data for stage classification limits the comparability with international studies in this field that used cancer registry data. It should also be noted here that the proportion of Death Certificate Only cases and missing stage information in German cancer registry data is approximately 30% for LC [1] which means that in Germany this data source would also be subject to uncertainties regarding stage classification.

In conclusion, our study shows that LC patients with more frequent healthcare utilization in the 3 years before LC diagnosis are more likely to be diagnosed at a non‐advanced stage. Regarding future LC screening in Germany, the findings underline the importance to ensure the reach of those who use health services less frequently. Implementation strategies that use health claims data to specifically identify these individuals could represent interesting options worth exploring as they are at higher risk of being diagnosed at an advanced stage if they develop LC.

## Author Contributions

J.K.‐K. contributed to the study design and analysis, interpreted the data and drafted the manuscript, supervised by U.H. and O.R. interpreted the data and critically revised the manuscript. U.H. contributed to the study design and analysis, interpreted the data and critically revised the manuscript. All authors approved the final draft for submission.

## Ethics Statement

In Germany, the utilization of health insurance data for scientific research is regulated by the Code of Social Law. All involved health insurance providers as well as the German Federal Office for Social Security and the Senator for Health, Women and Consumer Protection in Bremen as their responsible authorities approved the use of GePaRD data for this study. Informed consent for studies based on claims data is required by law unless obtaining consent appears unacceptable and would bias results, which was the case in this study. According to the Ethics Committee of the University of Bremen, studies based on GePaRD are exempt from institutional review board review.

## Conflicts of Interest

Josephine Küsel‐Kanbach, Oliver Riedel and Ulrike Haug are working at an independent, non‐profit research institute, the Leibniz Institute for Prevention Research and Epidemiology – BIPS. Unrelated to this study, BIPS occasionally conducts studies financed by the pharmaceutical industry. These are post‐authorization safety studies (PASS) requested by health authorities. The design and conduct of these studies as well as the interpretation and publication are not influenced by the pharmaceutical industry. The study presented was not funded by the pharmaceutical industry and was performed in line with the ENCePP Code of Conduct.

## Supporting information


**Table S1:** Patient characteristics stratified by stage at diagnosis (advanced vs. non‐advanced) and sex.
**Table S2:** Patient characteristics by stage using a modified stage classification (see methods section).
**Table S3:** Outpatient contacts and hospitalizations in the 3 years before LC diagno sis by stage using a modified stage classification (see methods section).
**Table S4:** Participation in preventive measures before LC diagnosis by stage using a modified stage classification (see methods section).
**Table S5:** Comorbidities of LC patients present before LC diagnosis, stratified by stage at diagnosis (advanced vs. non‐advanced) using a modified stage classification (see methods section).
**Table S6:** Outpatient contacts and hospitalizations in the 3 years before LC diagno sis stratified by stage and age group.
**Table S7:** Utilization of preventive measures before LC diagnosis stratified by stage and age group.
**Table S8:** Comorbidities of LC patients present before LC diagnosis, stratified by stage at diagnosis (advanced vs. non‐advanced) and age group.
**Table S9:** Comparison of crude and age‐adjusted odds ratios regarding the association between stage (advanced vs. non‐advanced) and outpatient contacts, hospitalizations as well as the uptake of preventive measures.
**Table S10:** Comparison of crude and age‐adjusted odds ratios regarding the association between stage (advanced vs. non‐advanced) and comorbidities.

## Data Availability

Access to the data is granted only to the employees on the BIPS premises and in the context of approved research projects. Access to the de‐identified dataset of this study is possible in cooperation with BIPS and after signing an agreement for guest researchers at BIPS on reasonable request.
