# Supplementary material for: Comparison of Healthcare Utilization Before Diagnosis Between Non‐Advanced and Advanced Lung Cancer Patients: A Study Among 72,851 Cases in Germany
Source: Int J Cancer. 2026 Aug 27;159(11):2657–64. doi: 10.1002/ijc.70682 (PMC13637057; doi:10.1002/ijc.70682)
Supplement: Supplementary file 1 [file IJC-159-2657-s001.pdf]

Supplementary materials

Comparison of healthcare utilization before diagnosis between non-advanced and advanced lung cancer patients: A study among 72,851 cases in Germany

Josephine Küsel-Kanbach, Oliver Riedel, Ulrike Haug

Contents

Supplementary table S1: Patient characteristics stratified by stage at diagnosis (advanced vs. non-advanced) and sex.

Supplementary table S2: Patient characteristics by stage using a modified stage classification (see methods section).

Supplementary table S3: Outpatient contacts and hospitalizations in the three years before LC diagnosis by stage using a modified stage classification (see methods section).

Supplementary table S4: Participation in preventive measures before LC diagnosis by stage using a modified stage classification (see methods section).

Supplementary table S5: Comorbidities of LC patients present before LC diagnosis, stratified by stage at diagnosis (advanced vs. non-advanced) using a modified stage classification (see methods section).

Supplementary table S6: Outpatient contacts and hospitalizations in the three years before LC diagnosis stratified by stage and age group.

Supplementary table S7: Utilization of preventive measures before LC diagnosis stratified by stage and age group.

Supplementary table S8: Comorbidities of LC patients present before LC diagnosis, stratified by stage at diagnosis (advanced vs. non-advanced) and age group.

Supplementary table S9: Comparison of crude and age-adjusted odds ratios regarding the association between stage (advanced vs. non-advanced) and outpatient contacts, hospitalizations as well as the uptake of preventive measures.

Supplementary table S10: Comparison of crude and age-adjusted odds ratios regarding the association between stage (advanced vs. non-advanced) and comorbidities.

Supplementary table S1: Patient characteristics stratified by stage at diagnosis (advanced vs. non-advanced) and sex.

		Women (n = 30,862)		Men (n = 41,989)	
		Non-advanced	Advanced	Non-advanced	Advanced
Total, n		8,842	22,020	11,722	30,267
Age at diagnosis					
	Mean (SD)	69.8 (11.1)	67.7 (10.5)	70.7 (10.4)	68.6 (10.2)
	Median (IQR)	70.0 (63.0 – 77.0)	68.0 (60.0 – 75.0)	72.0 (64.0 – 78.0)	69.0 (61.0 – 76.0)
GISD, n %					
	Low	1,676 (19.0)	4,219 (19.2)	1,811 (15.5)	4,756 (15.7)
	Medium	5,720 (64.7)	14,239 (64.6)	7,732 (66.0)	20,358 (67.3)
	High	1,439 (16.3)	3,557 (16.2)	2,173 (18.5)	5,144 (17.0)
	Missing	7 (<0.1)	5 (<0.1)	6 (<0.1)	9 (<0.1)
Age group, n (%)					
	<55 years	735 (8.3)	2,369 (10.8)	712 (6.1)	2,662 (8.8)
	55 - <65 years	1,971 (22.3)	6,129 (27.8)	2,527 (21.5)	7,897 (26.1)
	65 - <75 years	2,994 (33.9)	7,373 (33.5)	3,751 (32.0)	10,264 (33.9)
	≥75 years	3,142 (35.5)	6,149 (27.9)	4,732 (40.4)	9,444 (31.2)

GISD: German Index of Socioeconomic Deprivation. Quintiles classified into three categories: Low deprivation (1st quintile), medium deprivation (2nd – 4th quintile) and high deprivation (5th quintile). The GISD was assigned based on the district of residence. SD: Standard deviation. IQR: Range from the lower to the upper quartile.

Supplementary table S2: Patient characteristics by stage using a modified stage classification (see methods section).

		Women (n = 30,862)		Men (n = 41,989)	
		Non-advanced	Advanced	Non-advanced	Advanced
Total, n		7,534	23,328	9,673	32,316
Age at diagnosis					
	Mean (SD)	68.7 (10.9)	68.2 (10.7)	69.9 (10.3)	69.0 (10.3)
	Median (IQR)	70.0 (62.0 – 76.0)	68.0 (61.0 – 76.0)	71.0 (63.0 – 77.0)	69.0 (62.0 – 77.0)
GISD, n (%)					
	Low	1,423 (18.9)	4,472 (19.2)	1,492 (15.4)	5,075 (15.7)
	Medium	4,888 (64.9)	15,071 (64.6)	6,406 (66.2)	21,684 (67.1)
	High	1,216 (16.1)	3,780 (16.2)	1,771 (18.3)	5,546 (17.2)
	Missing	7 (0.1)	5 (<0.1)	4 (<0.1)	11 (<0.1)
Age group, n (%)					
	<55 years	701 (9.3)	2,403 (10.3)	670 (6.9)	2,704 (8.4)
	55 - <65 years	1,821 (24.2)	6,279 (26.9)	2,233 (23.1)	8,191 (25.4)
	65 - <75 years	2,628 (34.9)	7,739 (33.2)	3,919 (33.0)	10,824 (33.5)
	≥75 years	2,384 (31.6)	6,907 (29.6)	3,579 (37.0)	10,597 (32.8)

GISD: German Index of Socioeconomic Deprivation. Quintiles classified into three categories: Low deprivation (1st quintile), medium deprivation (2nd – 4th quintile) and high deprivation (5th quintile). The GISD was assigned based on the district of residence. SD: Standard deviation. IQR: Range from the lower to the upper quartile.

Supplementary table S3: Outpatient contacts and hospitalizations in the three years before LC diagnosis by stage using a modified stage classification (see methods section).

	Women			Men		
	Non-advanced n = 7,534	Advanced n = 23,328	Relative difference[1]	Non-advanced n = 9,673	Advanced n = 32,316	Relative difference[1]
**Outpatient contacts**						
At least one outpatient contact, n (%)	7,005 (93.0)	21,924 (94.0)	0.99	8,737 (90.3)	29,742 (92.0)	0.98
95% CI	92.4-93.5	93.7-94.3		89.7-90.9	91.7-92.3	
Among patients with at least one outpatient contact						
Mean (SD)	40.6 (22.0)	34.4 (20.3)	1.18	35.7 (20.9)	30.9 (19.8)	1.16
Median (IQR)	37.0 (25.0–53.0)	31.0 (20.0–45.0)	1.19	33.0 (21.0–47.0)	28.0 (17.0–42.0)	1.18
**Hospitalizations**						
At least one hospitalization, n (%)	5,223 (69.3)	13,031 (55.9)	1.24	6,781 (70.1)	18,928 (58.6)	1.20
95% CI	68.3-70.4	55.2-56.5		69.2-71.0	58.0-59.1	
Among patients with at least one hospitalization						
Mean (SD)	3.0 (2.8)	2.6 (2.5)	1.15	3.2 (2.9)	2.9 (2.8)	1.10
Median (IQR)	2.0 (1.0-4.0)	2.0 (1.0–3.0)	1.00	2.0 (1.0–4.0)	2.0 (1.0–4.0)	1.00
Duration of hospital stays in days						
Mean (SD)	27.2 (50.8)	25.5 (45.0)	1.07	27.4 (44.4)	25.4 (43.0)	1.08
Median (IQR)	14.0 (6.0–31.0)	12.0 (5.0–29.0)	1.17	15.0 (7.0–33.0)	13.0 (5.0–29.0)	1.15

[1]The relative difference is calculated as the proportion, median or mean among non-advanced LC patients divided by the proportion, median or mean among advanced LC patients, respectively.
CI: Confidence interval. SD: Standard deviation. IQR: Range from the lower to the upper quartile.

Supplementary table S4: Participation in preventive measures before LC diagnosis by stage using a modified stage classification (see methods section).

	Women			Men		
	Non-advanced n = 7,534	Advanced n = 23,328	Relative difference[5]	Non-advanced n = 9,673	Advanced n = 32,316	Relative difference[5]
	% (95% CI)	% (95% CI)	Ratio	% (95% CI)	% (95% CI)	Ratio
General health check-up ("Check-up 35")[1]	61.8 (60.7–62.9)	60.2 (59.6–60.8)	1.03	58.2 (57.2–59.2)	57.4 (56.9–57.9)	1.01
Mammography screening[1]	26.8 (25.8–27.9)	25.9 (25.3–26.4)	1.03	-	-	-
Gynecological examination[1,3]	45.0 (43.8–46.1)	40.6 (39.9–41.2)	1.11	-	-	-
Urological examination[1,4]	-	-	-	40.5 (39.5–41.5)	36.5 (36.0–37.0)	1.11
Skin cancer screening[1]	39.7 (38.6–40.8)	37.4 (36.7–38.0)	1.06	38.1 (37.1–39.0)	36.2 (35.6–36.7)	1.05
Colonoscopy screening[2]	16.1 (15.2–16.9)	13.8 (13.4–14.3)	1.17	15.7 (15.0–16.4)	13.6 (13.3–14.0)	1.15
Fecal Occult Blood Test (FOBT)[1]	21.8 (20.9–22.8)	20.7 (20.1–21.2)	1.05	19.1 (18.4–19.9)	18.1 (17.7–18.5)	1.06
Screening for abdominal aortic aneurysm[2]	-	-	-	2.9 (2.6–3.3)	2.7 (2.5–2.9)	1.07

[1] Three years prior to LC diagnosis
[2] Ever before the LC diagnosis.
[3] The reimbursement code "gynecological examination" includes early detection of cervical cancer by cytology and breast palpation.
[4] This reimbursement code includes digital rectal examination of the prostate, examination of external genitalia and query of medical history.
[5] The relative difference is calculated as the proportion among non-advanced LC patients divided by the proportion among advanced LC patients.
CI: Confidence interval.

Supplementary table S5: Comorbidities of LC patients present before LC diagnosis, stratified by stage at diagnosis (advanced vs. non-advanced) using a modified stage classification (see methods section).

	Women					Men				
	Non-advanced n = 7,534		Advanced n = 23,328		Relative difference[2]	Non-advanced n = 9,673		Advanced n = 32,316		Relative difference[2]
	%	95% CI	%	95% CI	Ratio	%	95% CI	%	95% CI	Ratio
Comorbidities										
Coronary heart disease	20.3	19.4–21.2	16.6	16.2–17.1	1.22	33.7	32.7–34.6	29.1	28.6–29.6	1.16
Congestive heart failure	11.4	10.7-12.1	9.8	9.4–10.2	1.16	15.1	14.4–15.8	13.0	12.6–13.3	1.16
Myocardial infarction	3.6	3.2–4.0	3.0	2.8–3.2	1.20	7.3	6.8–7.8	6.3	6.0–6.6	1.16
Stroke	4.4	4.0–4.9	4.1	3.9–4.4	1.07	5.9	5.5–6.4	6.1	5.8–6.3	0.97
Platelet aggregation inhibitors	6.6	6.1–7.2	5.8	5.5–6.1	1.14	12.7	12.1–13.4	11.0	10.7–11.4	1.15
Treatment with acetylsalicylic acid	13.6	12.8–14.3	12.0	11.6–12.4	1.13	22.5	21.6–23.3	20.4	19.9–20.8	1.10
Dual antiplatelet therapy	3.3	2.9–3.7	2.9	2.7–3.1	1.14	6.7	6.2–7.2	6.0	5.7–6.2	1.12
Treatment with anticoagulant therapy	9.5	8.9–10.2	7.6	7.2–7.9	1.25	13.7	13.1–14.4	12.4	12.0–12.7	1.10
Treatment with lipid-modifying agents	28.6	27.6–29.6	25.8	25.2–26.3	1.11	36.3	35.4–37.3	33.5	33.0–34.0	1.08
Drug-treated hypertension	57.3	56.2–58.4	54.1	53.5–54.7	1.06	60.0	59.1–61.0	57.1	56.6–57.7	1.05
COPD	37.8	36.7–38.9	29.7	29.1–30.3	1.27	37.0	36.1–38.0	27.5	27.0–28.0	1.35
Treated bronchial asthma	13.6	12.9–14.4	9.3	9.0–9.7	1.46	8.7	8.2–9.3	5.6	5.4–5.9	1.55
Liver diseases including chronic viral hepatitis	16.9	16.1–17.7	16.2	15.7–16.6	1.04	20.1	19.3–20.9	19.2	18.8–19.7	1.05
Treated diabetes	12.0	11.3–12.7	11.5	11.1–11.9	1.04	17.9	17.1–18.6	17.9	17.4–18.3	1.00
Treatment with immunosuppressive drugs	14.5	13.7–15.3	11.0	10.6–11.4	1.32	11.6	11.0–12.3	8.8	8.5–9.1	1.32
Hemiplegia	4.7	4.3–5.2	4.0	3.7–4.2	1.18	5.1	4.7–5.6	5.1	4.8–5.3	1.00
At least two of the above[3]	44.7	43.6-45.8	36.2	35.6-36.9	1.23	53.5	52.5-54.5	46.2	45.6-46.7	1.16
Indicators of unhealthy lifestyle										
Alcohol abuse	9.8	9.1–10.4	10.2	9.9–10.6	0.96	17.8	17.1–18.6	17.7	17.2–18.1	1.01
Smoking[1]	44.6	43.5–45.8	45.0	44.3–45.6	0.99	46.2	45.2–47.2	44.4	43.9–45.0	1.04
Obesity	28.8	27.8–29.9	26.7	26.1–27.2	1.08	26.7	25.9–27.6	26.2	25.7–26.7	1.02

[1] Information on smoking is obtained from the claims data via information on mental and behavioral disorders due to use of tobacco (ICD-10 GM: F17), tobacco or nicotine poisoning (T65.2) or by prescribing medication to treat nicotine dependence (ATC-Code: N07BA). As information on smoking is not routinely collected, the proportion of smokers is likely underestimated in German health claims data.

[2]The relative difference is calculated as the proportion among non-advanced LC patients divided by the proportion among advanced LC patients.

CI: Confidence interval, COPD: Chronic obstructive pulmonary disease.

[3]Less severe comorbidities (treatment with lipid-modifying agents and drug-treated hypertension) were not considered here.

Supplementary table S6: Outpatient contacts and hospitalizations in the three years before LC diagnosis stratified by stage and age group.

	Women (n = 30,862)			Men (n = 41,989)		
	Non-advanced	Advanced	Relative difference[1]	Non-advanced	Advanced	Relative difference[1]
Age at diagnosis						
< 55 years						
**Outpatient contacts**						
At least one outpatient contact, n (%)	693 (94.3)	2,236 (94.4)	1.00	645 (90.6)	2,419 (90.9)	1.00
95% CI	92.4-95.7	93.4-95.2		88.2-92.5	89.7-91.9	
Among patients with at least one outpatient contact						
Mean (SD)	35.3 (21.5)	28.6 (17.9)	1.23	24.7 (18.3)	19.6 (15.4)	1.26
Median (IQR)	32.0 (21.0–45.0)	26.0 (16.0–38.0)	1.23	22.0 (11.0–33.0)	16.0 (8.0–27.0)	1.38
**Hospitalizations**						
At least one hospitalization, n (%)	453 (61.6)	1,097 (46.3)	1.33	452 (63.5)	1,240 (46.6)	1.36
95% CI	58.1-65.1	44.3-48.3		59.9-66.9	44.7-48.5	
Among patients with at least one hospitalization						
Mean (SD)	3.0 (3.1)	2.3 (2.4)	1.30	2.9 (2.9)	2.8 (3.2)	1.04
Median (IQR)	2.0 (1.0–4.0)	2.0 (1.0–3.0)	1.00	2.0 (1.0–4.0)	2.0 (1.0–3.0)	1.00
Duration of hospital stays in days						
Mean (SD)	24.7 (47.1)	20.8 (44.5)	1.19	26.9 (62.8)	27.0 (55.1)	1.00
Median (IQR)	10.0 (4.0–24.0)	8.0 (4.0–20.0)	1.25	11.0 (5.0–27.0)	9.0 (4.0–27.5)	1.22
55 - 64 years						
**Outpatient contacts**						
At least one outpatient contact, n (%)	1,821 (92.4)	5,757 (93.9)	0.98	2,293 (90.7)	7,244 (91.7)	0.99
95% CI	91.1-93.5	93.3-94.5		89.5-91.8	91.1-92.3	
Among patients with at least one outpatient contact						
Mean (SD)	38.2 (22.2)	32.1 (20.1)	1.19	29.6 (19.1)	24.6 (17.1)	1.20
Median (IQR)	35.0 (23.0–50.0)	28.0 (18.0–43.0)	1.25	26.0 (16.0–40.0)	22.0 (12.0–34.0)	1.18
**Hospitalizations**						
At least one hospitalization, n (%)	1,236 (62.7)	3,002 (49.0)	1.28	1,654 (65.5)	4,142 (52.5)	1.25
95% CI	60.5-64.8	47.7-50.2		63.6-67.3	51.3-53.5	
Among patients with at least one hospitalization						
Mean (SD)	3.0 (2.9)	2.5 (2.4)	1.20	3.3 (3.2)	2.7 (2.9)	1.22
Median (IQR)	2.0 (1.0–4.0)	2.0 (1.0–3.0)	1.00	2.0 (1.0–4.0)	2.0 (1.0–3.0)	1.00
Duration of hospital stays in days						
Mean (SD)	29.8 (66.7)	22.9 (43.1)	1.30	29.0 (43.0)	23.8 (42.2)	1.22
Median (IQR)	12.0 (5.0–30.0)	10.0 (4.0–23.0)	1.20	15.0 (6.0–34.0)	11.0 (4.0–26.0)	1.36
65 - 74 years						
**Outpatient contacts**						
At least one outpatient contact, n (%)	2,783 (93.0)	6,907 (93.7)	0.99	3,375 (90.0)	9,446 (92.0)	0.98
95% CI	92.0-93.8	93.1-94.2		89.0-90.9	91.5-92.5	
Among patients with at least one outpatient contact						
Mean (SD)	40.8 (22.2)	35.5 (20.7)	1.15	35.1 (20.5)	31.0 (18.8)	1.13
Median (IQR)	38.0 (25.0–53.0)	33.0 (21.0–47.0)	1.15	32.0 (21.0–46.0)	28.0 (17.0–42.0)	1.14
**Hospitalizations**						
At least one hospitalization, n (%)	2,067 (69.0)	4,014 (54.4)	1.27	2,578 (68.7)	5,949 (58.0)	1.18
95% CI	67.4-70.7	53.3-55.6		67.2-70.2	57.0-58.9	
Among patients with at least one hospitalization						
Mean (SD)	3.0 (2.6)	2.7 (2.6)	1.11	3.2 (2.8)	2.8 (2.7)	1.14
Median (IQR)	2.0 (1.0–4.0)	2.0 (1.0–3.0)	1.00	2.0 (1.0–4.0)	2.0 (1.0–3.0)	1.00

Duration of hospital stays in days						
Mean (SD)	27.9 (58.0)	24.6 (45.5)	1.13	30.0 (54.9)	23.1 (36.9)	1.30
Median (IQR)	13.0 (6.0–29.0)	12.0 (5.0–27.0)	1.08	15.0 (7.0–35.0)	12.0 (5.0–26.0)	1.25
≥ 75 years						
**Outpatient contacts**						
At least one outpatient contact, n (%)	2,924 (93.1)	5,808 (94.5)	0.99	4,283 (90.5)	8,774 (92.9)	0.97
95% CI	92.1-93.9	93.8-95.0		89.6-91.3	92.4-93.4	
Among patients with at least one outpatient contact						
Mean (SD)	40.8 (21.4)	37.4 (20.1)	1.09	40.8 (21.2)	38.3 (20.4)	1.07
Median (IQR)	37.0 (26.0–53.0)	35.0 (23.0–48.0)	1.06	38.0 (26.0–53.0)	36.0 (24.0–49.0)	1.06
**Hospitalizations**						
At least one hospitalization, n (%)	2,371 (75.5)	4,014 (65.3)	1.16	3,513 (74.2)	6,181 (65.5)	1.13
95% CI	73.9-76.9	64.1-66.5		73.0-75.5	64.5-66.4	
Among patients with at least one hospitalization						
Mean (SD)	3.1 (2.7)	2.7 (2.3)	1.15	3.2 (2.8)	2.9 (2.6)	1.10
Median (IQR)	2.0 (1.0–4.0)	2.0 (1.0–3.0)	1.00	2.0 (1.0 – 4.0)	2.0 (1.0–4.0)	1.00
Duration of hospital stays in days						
Mean (SD)	30.0 (36.6)	26.7 (41.8)	1.12	28.5 (40.2)	25.9 (41.8)	1.10
Median (IQR)	18.0 (8.0–38.0)	15.0 (7.0–33.0)	1.20	16.0 (8.0 – 35.0)	15.0 (6.0–31.0)	1.07

[1]The relative difference is calculated as the proportion, median or mean among non-advanced LC patients divided by the proportion, median or mean among advanced LC patients, respectively.
CI: Confidence interval. SD: Standard deviation. IQR: Range from the lower to the upper quartile.

Supplementary table S7: Utilization of preventive measures before LC diagnosis stratified by stage and age group.

	Women (n = 30,862)			Men (n = 41,989)		
	Non-advanced	Advanced	Relative difference[5]	Non-advanced	Advanced	Relative difference[5]
	% (95% CI)	% (95% CI)	Ratio	% (95% CI)	% (95% CI)	Ratio
Age at diagnosis						
<55 years						
General health check-up ("Check-up 35")[1]	55.2 (51.6–58.8)	53.9 (51.9–55.9)	1.02	47.5 (43.8–51.1)	47.1 (45.2–49.0)	1.01
Mammography screening[1]	22.7 (19.8–25.9)	23.4 (21.7–25.1)	0.97	-	-	-
Gynecological examination[1,3]	66.7 (63.2–70.0)	60.2 (58.2–62.1)	1.11	-	-	-
Urological examination[1,4]	-	-	-	22.1 (19.2–25.2)	19.1 (17.7–20.7)	1.16
Skin cancer screening[1]	35.9 (32.5–39.5)	34.1 (32.2–36.0)	1.05	31.6 (28.3–35.1)	28.0 (26.3–29.7)	1.13
Colonoscopy screening[2]	0.0 (0.0–0.5)	0.1 (0.0–0.3)	0.00	0.1 (0.0–0.8)	0.0 (0.0–0.1)	0.00
Fecal Occult Blood Test (FOBT)[1]	19.7 (17.0–22.8)	18.6 (17.1–20.2)	1.06	5.8 (4.3–7.7)	6.2 (5.3–7.2)	0.94
Screening for abdominal aortic aneurysm[2]	-	-	-	0.0 (0.0–0.5)	0.0 (0.0–0.1)	0.00
55 - 64 years						
General health check-up ("Check-up 35")[1]	59.7 (57.5–61.8)	58.6 (57.3–59.8)	1.02	56.4 (54.4–58.3)	54.5 (53.4–55.6)	1.03
Mammography screening[1]	47.7 (45.5–49.9)	46.4 (45.1–47.6)	1.03	-	-	-
Gynecological examination[1,3]	47.7 (45.5–49.9)	45.9 (44.7–47.2)	1.04	-	-	-
Urological examination[1,4]	-	-	-	33.4 (31.6–35.3)	30.3 (29.3–31.4)	1.10
Skin cancer screening[1]	37.8 (35.7–40.0)	35.9 (34.7–37.1)	1.05	34.9 (33.0–36.7)	32.3 (31.3–33.3)	1.08
Colonoscopy screening[2]	11.5 (10.1–12.9)	10.0 (9.3–10.8)	1.15	8.8 (7.7–10.0)	8.4 (7.8-9.0)	1.05
Fecal Occult Blood Test (FOBT)[1]	25.7 (23.8–27.6)	25.9 (24.8–27.0)	0.99	16.5 (15.1–18.0)	15.6 (14.8–16.4)	1.06
Screening for abdominal aortic aneurysm[2]	-	-	-	0.0 (0.0–0.2)	0.0 (0.0–0.1)	0.00
65 - 74 years						
General health check-up ("Check-up 35")[1]	62.6 (60.8–64.3)	62.0 (60.9–63.1)	1.01	58.9 (57.3–60.5)	59.1 (58.2–60.1)	1.00
Mammography screening[1]	34.4 (32.8–36.2)	34.2 (33.1–35.3)	1.01	-	-	-
Gynecological examination[1,3]	44.1 (42.3–45.8)	40.4 (39.3–41.6)	1.09	-	-	-
Urological examination[1,4]	-	-	-	40.7 (39.2–42.3)	38.7 (37.8–39.6)	1.05
Skin cancer screening[1]	39.5 (37.7–41.2)	39.1 (38.0–40.2)	1.01	37.5 (35.9–39.0)	37.3 (36.4–38.3)	1.01
Colonoscopy screening[2]	21.1 (19.7–22.6)	19.0 (18.2–20.0)	1.11	18.1 (16.9–19.3)	17.4 (16.7–18.1)	1.04
Fecal Occult Blood Test (FOBT)[1]	22.1 (20.7–23.7)	20.9 (20.0–21.8)	1.06	19.8 (18.5–21.1)	19.8 (19.0–20.6)	1.00
Screening for abdominal aortic aneurysm[2]	-	-	-	3.3 (2.8–3.9)	3.4 (3.1–3.8)	0.97
75 years						
General health check-up ("Check-up 35")[1]	61.1 (59.4–62.8)	63.1 (61.9–64.3)	0.97	58.3 (56.9–59.7)	61.7 (60.7–62.7)	0.94
Mammography screening[1]	0.1 (0.0–0.3)	0.0 (0.0–0.1)	0.00	-	-	-
Gynecological examination[1,3]	29.7 (28.1-31.3)	31.7 (30.5–32.8)	0.94	-	-	-
Urological examination[1,4]	-	-	-	43.5 (42.1–44.9)	45.0 (44.0–46.0)	0.97
Skin cancer screening[1]	38.2 (36.5-39.9)	39.5 (38.3–40.8)	0.97	38.6 (37.3–40.0)	41.3 (40.3–42.3)	0.93
Colonoscopy screening[2]	16.1 (14.9–17.4)	17.1 (16.2–18.1)	0.94	17.9 (16.8–19.0)	18.3 (17.5–19.1)	0.98
Fecal Occult Blood Test (FOBT)[1]	16.0 (14.8–17.3)	17.6 (16.6-18.5)	0.91	20.4 (19.2–21.5)	22.4 (21.5–23.2)	0.91
Screening for abdominal aortic aneurysm[2]	-	-	-	4.4 (3.9–5.0)	5.1 (4.6–5.5)	0.86

[1] Three years prior to LC diagnosis

[2] Ever before the LC diagnosis.

[3] The reimbursement code "gynecological examination" includes early detection of cervical cancer by cytology and breast palpation.

[4] This reimbursement code includes digital rectal examination of the prostate, examination of external genitalia and query of

medical history.
[5] The relative difference is calculated as the proportion among non-advanced LC patients divided by the proportion among advanced LC patients.
CI: Confidence interval.

Supplementary table S8: Comorbidities of LC patients present before LC diagnosis, stratified by stage at diagnosis (advanced vs. non-advanced) and age group.

	Women (n = 30,862)					Men (n = 41,989)				
	Non-advanced		Advanced		Relative difference[2]	Non-advanced		Advanced		Relative difference[2]
	%	95% CI	%	95% CI	Ratio	%	95% CI	%	95% CI	Ratio
Age at diagnosis										
<55 years										
Comorbidities										
Coronary heart disease	4.8	3.4–6.6	3.0	2.4–3.8	1.60	8.7	6.9–11.0	6.8	5.9–7.8	1.28
Congestive heart failure	2.7	1.8–4.2	0.9	0.6–1.4	3.00	3.1	2.0–4.6	1.9	1.5–2.5	1.63
Myocardial infarction	1.4	0.7–2.5	0.8	0.5–1.3	1.75	3.4	2.3–5.0	2.5	2.0–3.2	1.36
Stroke	1.4	0.7–2.5	1.1	0.8–1.6	1.27	2.5	1.6–4.0	1.5	1.1–2.1	1.67
Platelet aggregation inhibitors	2.4	1.6–3.8	1.2	0.9–1.8	2.08	3.7	2.5–5.3	2.9	2.4–3.6	1.28
Treatment with acetylsalicylic acid	4.8	3.4–6.6	2.5	2.0–3.2	1.92	6.6	5.0–8.7	5.1	4.3–6.0	1.29
Dual antiplatelet therapy	1.5	0.8–2.7	0.7	0.4–1.1	2.14	2.1	1.3–3.4	2.0	1.5–2.6	1.05
Treatment with anticoagulant therapy	2.0	1.2–3.3	1.5	1.1-2.0	1.33	2.8	1.8–4.3	2.0	1.5–2.6	1.40
Treatment with lipid-modifying agents	5.9	4.4–7.8	5.4	4.5–6.3	1.09	11.1	9.0–13.6	9.2	8.1–10.3	1.21
Drug-treated hypertension	23.4	20.5–26.6	20.9	19.3–22.6	1.12	26.0	22.9–29.3	21.4	19.9–23.0	1.21
COPD	27.4	24.2–30.7	18.4	16.9–20.1	1.49	20.6	17.8–23.8	14.7	13.4–16.1	1.41
Treated bronchial asthma	13.3	11.1–16.0	9.0	7.9–10.3	1.48	7.0	5.4–9.1	3.8	3.2–4.6	1.84
Liver diseases including chronic viral hepatitis	11.2	9.1–13.6	8.4	7.3–9.5	1.33	16.4	13.9–19.3	13.1	11.9–14.5	1.24
Treated diabetes	5.2	3.8-7.0	3.2	2.5–4.0	1.63	8.0	6.2–10.2	5.9	5.0–6.8	1.36
Treatment with immunosuppressive drugs	12.0	9.8–14.5	7.6	6.6–8.8	1.58	8.4	6.6–10.7	4.6	3.9–5.4	1.83
Hemiplegia	2.9	1.9–4.3	1.8	1.3–2.4	1.61	3.7	2.5–5.3	2.5	2.0–3.2	1.48
At least two of the above[3]	24.1	21.1-27.3	13.8	12.5-15.3	1.75	24.3	21.3-27.6	15.9	14.6-17.4	1.53
Indicators of unhealthy lifestyle										
Alcohol abuse	12.1	9.9–14.7	9.6	8.5–10.9	1.26	24.4	21.4–27.7	23.4	21.8–25.0	1.04
Smoking[1]	44.5	40.9–48.1	48.0	46.0–50.1	0.93	46.8	43.1–50.4	46.0	44.1–47.9	1.02
Obesity	23.7	20.7–26.9	19.5	18.0–21.2	1.22	17.7	15.1–20.7	16.1	14.7–17.5	1.10
55 - 64 years										
Comorbidities										
Coronary heart disease	10.9	9.6–12.4	8.9	8.3–9.7	1.22	20.2	18.7–21.8	17.6	16.8–18.5	1.15
Congestive heart failure	5.1	4.2–6.2	3.9	3.4–4.4	1.31	9.1	8.0–10.3	5.8	5.3–6.3	1.57
Myocardial infarction	2.1	1.5–2.8	1.8	1.5–2.2	1.17	5.4	4.6–6.3	5.2	4.7–5.7	1.04
Stroke	3.3	2.6–4.2	2.9	2.5–3.4	1.14	4.2	3.5–5.0	3.6	3.2–4.1	1.17
Platelet aggregation inhibitors	3.8	3.0–4.7	3.4	3.0–3.9	1.12	9.3	8.2–10.5	7.4	6.8–8.0	1.26

Treatment with acetylsalicylic acid	8.6	7.4–9.9	6.7	6.1–7.3	1.28	16.7	15.3–18.2	14.6	13.8–15.4	1.14
Dual antiplatelet therapy	2.1	1.6–2.9	1.9	1.6–2.2	1.11	5.7	4.8–6.6	4.6	4.2–5.1	1.24
Treatment with anticoagulant therapy	4.9	4.0–5.9	2.5	2.1–2.9	1.96	6.4	5.5–7.4	5.3	4.8–5.8	1.21
Treatment with lipid-modifying agents	20.5	18.8–22.4	16.4	15.5–17.3	1.25	25.1	23.5–26.9	23.1	22.2–24.1	1.09
Drug-treated hypertension	44.5	42.3–46.7	40.6	39.3–41.8	1.10	47.5	45.5–49.4	43.2	42.1–44.3	1.10
COPD	37.9	35.8–40.1	27.3	26.2–28.5	1.39	35.5	33.6–37.3	23.1	22.2–24.1	1.54
Treated bronchial asthma	13.7	12.3–15.3	8.8	8.1–9.5	1.56	9.1	8.0–10.2	4.8	4.4–5.3	1.90
Liver diseases including chronic viral hepatitis	15.5	13.9–17.1	14.2	13.3–15.1	1.09	21.1	19.5–22.7	18.3	17.5–19.2	1.15
Treated diabetes	9.2	8.0–10.5	8.2	7.5–8.9	1.12	13.2	12.0-14.6	12.6	11.9–13.4	1.05
Treatment with immunosuppressive drugs	14.1	12.6–15.7	9.6	8.9–10.4	1.47	10.7	9.5–11.9	7.2	6.7–7.8	1.49
Hemiplegia	4.9	4.0–5.9	3.4	3.0–3.9	1.44	5.4	4.6–6.3	3.9	3.5–4.4	1.38
At least two of the above[3]	34.8	32.7-36.9	25.6	24.5-26.7	1.36	42.5	40.5-44.4	32.8	31.8-33.8	1.30
Indicators of unhealthy lifestyle										
Alcohol abuse	13.6	12.2–15.2	13.7	12.9–14.6	0.99	29.5	27.8–31.3	24.6	23.6–25.5	1.20
Smoking[1]	55.8	53.6–58.0	53.2	51.9–54.4	1.05	60.2	58.3–62.1	54.7	53.6–55.7	1.10
Obesity	28.7	26.7–30.7	23.8	22.7–24.9	1.21	23.0	21.4–24.7	22.9	22.0–23.9	1.00
65 - 74 years										
Comorbidities										
Coronary heart disease	20.6	19.2–22.1	16.5	15.7–17.4	1.25	34.4	32.9–36.0	29.8	28.9–30.7	1.15
Congestive heart failure	10.3	9.2–11.4	8.3	7.7–8.9	1.24	14.9	13.8–16.1	11.9	11.3–12.5	1.25
Myocardial infarction	3.3	2.8–4.0	3.5	3.1–3.9	0.94	7.6	6.8–8.5	6.4	6.0–6.9	1.19
Stroke	4.9	4.2–5.7	3.8	3.4–4.3	1.29	6.7	6.0–7.6	6.4	6.0–6.9	1.05
Platelet aggregation inhibitors	6.9	6.1–7.9	6.5	5.9–7.1	1.06	13.8	12.7–15.0	11.9	11.3–12.5	1.16
Treatment with acetylsalicylic acid	13.7	12.5–14.9	11.8	11.1–12.5	1.16	23.4	22.0–24.7	21.4	20.7–22.2	1.09
Dual antiplatelet therapy	3.4	2.8–4.1	3.1	2.7–3.5	1.10	7.4	6.6–8.3	6.2	5.8–6.7	1.19
Treatment with anticoagulant therapy	9.1	8.1–10.1	6.5	6.0–7.1	1.40	13.7	12.6–14.8	11.3	10.7–11.9	1.21
Treatment with lipid-modifying agents	30.4	28.8–32.1	29.1	28.1–30.2	1.04	38.7	37.2–40.3	37.1	36.2–38.0	1.04
Drug-treated hypertension	61.4	59.7–63.2	57.9	56.8–59.0	1.06	62.4	60.9–64.0	60.6	59.7–61.5	1.03
COPD	42.2	40.5–44.0	32.9	31.8–33.9	1.28	40.2	38.7–41.8	29.9	29.0–30.7	1.34
Treated bronchial asthma	14.7	13.5–16.0	10.0	9.4–10.7	1.47	8.8	7.9–9.7	5.8	5.4–6.3	1.52
Liver diseases including chronic viral hepatitis	18.8	17.5–20.3	17.9	17.0–18.8	1.05	20.9	19.6–22.2	20.9	20.1–21.7	1.00
Treated diabetes	13.7	12.5–15.0	12.7	11.9–13.4	1.08	20.7	19.5–22.1	20.0	19.2–20.8	1.04
Treatment with immunosuppressive drugs	15.5	14.3–16.9	11.5	10.8–12.2	1.35	11.9	10.9–13.0	8.6	8.0–9.1	1.38
Hemiplegia	4.6	3.9–5.5	4.0	3.6–4.4	1.15	5.5	4.9–6.3	5.1	4.7–5.5	1.08
At least two of the above[3]	48.1	46.3-49.9	38.2	37.1-39.3	1.26	56.4	54.8-58.0	48.3	47.3-49.3	1.17
Indicators of unhealthy lifestyle										

Alcohol abuse	11.9	10.8–13.1	10.4	9.8–11.2	1.14	19.9	18.7–21.3	17.8	17.1–18.5	1.12
Smoking[1]	51.6	49.8–53.4	49.4	48.3–50.6	1.04	51.9	50.3–53.5	49.6	48.7–50.6	1.05
Obesity	29.1	27.5–30.7	28.3	27.3–29.3	1.03	28.3	26.9–29.8	28.9	28.0–29.8	0.98
≥75 years										
Comorbidities										
Coronary heart disease	32.2	30.6–33.9	27.6	26.5–28.7	1.17	45.5	44.1–46.9	42.6	41.6–43.6	1.07
Congestive heart failure	23.2	21.8–24.7	18.2	17.3–19.2	1.27	24.3	23.1–25.6	20.7	19.9–21.6	1.17
Myocardial infarction	5.6	4.9–6.5	4.1	3.7–4.7	1.37	8.9	8.1–9.7	7.7	7.2–8.3	1.16
Stroke	7.0	6.2–8.0	6.0	5.5–6.7	1.17	8.3	7.6–9.2	8.2	7.6–8.7	1.01
Platelet aggregation inhibitors	10.2	9.2–11.4	8.3	7.7–9.1	1.23	15.8	14.8–16.9	14.7	14.0–15.4	1.07
Treatment with acetylsalicylic acid	22.0	20.6–23.5	19.2	18.2–20.2	1.15	28.9	27.6–30.2	27.0	26.1–27.9	1.07
Dual antiplatelet therapy	4.8	4.1–5.6	4.1	3.7–4.7	1.17	7.8	7.0–8.6	7.6	7.1–8.2	1.03
Treatment with anticoagulant therapy	17.7	16.4–19.1	14.3	13.4–15.2	1.24	22.2	21.0–23.4	20.6	19.8–21.5	1.08
Treatment with lipid-modifying agents	38.7	37.0–40.4	37.6	36.4–38.9	1.03	44.6	43.2–46.0	44.4	43.4–45.4	1.00
Drug-treated hypertension	74.3	72.8–75.8	72.5	71.4–73.6	1.02	73.1	71.8–74.4	72.9	72.0–73.8	1.00
COPD	36.6	34.9–38.3	30.5	29.3–31.6	1.20	37.5	36.1–38.9	30.2	29.3–31.1	1.24
Treated bronchial asthma	12.1	11.0–13.2	8.5	7.8–9.2	1.42	8.2	7.5–9.1	6.2	5.7–6.7	1.32
Liver diseases including chronic viral hepatitis	18.5	17.2–19.9	18.3	17.3–19.3	1.01	19.4	18.3–20.5	19.8	19.0–20.6	0.98
Treated diabetes	15.7	14.5–17.0	15.3	14.4–16.2	1.03	21.4	20.3–22.6	22.3	21.5–23.2	0.96
Treatment with immunosuppressive drugs	14.9	13.7–16.2	12.0	11.2–12.9	1.24	13.6	12.6–14.6	10.3	9.7–10.9	1.32
Hemiplegia	6.3	5.5–7.2	4.5	4.0–5.1	1.40	6.2	5.5–6.9	6.1	5.6–6.6	1.02
At least two of the above[3]	57.6	55.9-59.3	48.8	47.5-50.0	1.18	64.7	63.3-66.1	60.3	59.3-61.3	1.07
Indicators of unhealthy lifestyle										
Alcohol abuse	6.5	5.7–7.4	6.0	5.4–6.6	1.08	10.1	9.3–11.0	9.5	8.9–10.1	1.06
Smoking[1]	30.0	28.4–31.6	30.9	29.7–32.0	0.97	32.0	30.7–33.4	30.5	29.6–31.4	1.05
Obesity	30.9	29.3–32.6	29.3	28.1–30.4	1.05	28.3	27.0–29.6	29.0	28.1–29.9	0.98

[1] Information on smoking is obtained from the claims data via information on mental and behavioral disorders due to use of tobacco (ICD-10 GM: F17), tobacco or nicotine poisoning (T65.2) or by prescribing medication to treat nicotine dependence (ATC-Code: N07BA). As information on smoking is not routinely collected, the proportion of smokers is likely underestimated in German health claims data.

[2]The relative difference is calculated as the proportion among non-advanced LC patients divided by the proportion among advanced LC patients.

CI: Confidence interval, COPD: Chronic obstructive pulmonary disease.

[3]Less severe comorbidities (treatment with lipid-modifying agents and drug-treated hypertension) were not considered here.

Supplementary table S9: Comparison of crude and age-adjusted odds ratios regarding the association between stage (advanced vs. non-advanced) and outpatient contacts, hospitalizations as well as the uptake of preventive measures.

	Women (n = 30,862)				Men (n = 41,989)			
	Crude		Age-adjusted		Crude		Age-adjusted	
	OR	95% CI	OR	95% CI	OR	95% CI	OR	95% CI
Any outpatient contact[1]	0.84	0.76–0.93	0.84	0.76–0.93	0.80	0.75–0.87	0.79	0.74–0.85
Any hospitalization[1]	1.84	1.75–1.94	1.77	1.68–1.86	1.69	1.62–1.77	1.62	1.55–1.70
General health check-up (“Check-up 35”)[1]	1.01	0.96–1.07	0.99	0.95–1.05	0.99	0.95–1.03	0.96	0.92–1.00
Mammography Screening[1]	0.87	0.82–0.92	0.98	0.92–1.04	-	-	-	-
Gynecological examination[1,3]	1.00	0.95–1.05	1.08	1.03-1.14	-	-	-	-
Urological examination[1,4]	-	-	-	-	1.11	1.06–1.16	1.04	0.99–1.08
Skin cancer screening[1]	1.02	0.97–1.08	1.01	0.96–1.07	1.03	0.98–1.07	0.99	0.95–1.04
Colonoscopy screening[2]	1.13	1.05–1.21	1.05	0.98–1.12	1.10	1.03–1.16	1.00	0.94–1.07
Fecal Occult Blood Test (FOBT)[1]	0.97	0.91–1.03	0.99	0.93–1.05	1.01	0.96–1.07	0.95	0.90–1.01
Screening for abdominal aortic aneurysm[2]	-	-	-	-	1.04	0.91–1.18	0.89	0.78–1.01

[1] Three years prior to LC diagnosis
[2] Ever before the LC diagnosis.
[3] The reimbursement code “gynecological examination” includes early detection of cervical cancer by cytology and breast palpation.
[4] This reimbursement code includes digital rectal examination of the prostate, examination of external genitalia and query of medical history.
OR: Odds ratio, CI: Confidence interval.

Supplementary table S10: Comparison of crude and age-adjusted odds ratios regarding the association between stage (advanced vs. non-advanced) and comorbidities.

	Women (n = 30,862)				Men (n = 41,989)			
	Crude		Age-adjusted		Crude		Age-adjusted	
	OR	95% CI	OR	95% CI	OR	95% CI	OR	95% CI
Comorbidities								
Coronary heart disease	1.41	1.33–1.51	1.25	1.17–1.33	1.30	1.24–1.36	1.16	1.10–1.21
Congestive heart failure	1.52	1.40–1.64	1.29	1.19–1.40	1.45	1.37–1.54	1.28	1.20–1.36
Myocardial infarction	1.28	1.12–1.47	1.17	1.02–1.34	1.21	1.11–1.32	1.15	1.05-1.25
Stroke	1.30	1.15–1.46	1.18	1.05–1.33	1.14	1.05–1.25	1.05	0.96–1.15
Platelet aggregation inhibitors	1.28	1.16–1.41	1.17	1.05–1.29	1.24	1.16–1.32	1.15	1.07–1.22
Treatment with acetylsalicylic acid	1.34	1.25–1.44	1.19	1.11–1.29	1.21	1.15–1.27	1.11	1.05–1.17
Dual antiplatelet therapy	1.26	1.09–1.44	1.16	1.01–1.33	1.18	1.08–1.29	1.11	1.02–1.21
Treatment with anticoagulant therapy	1.57	1.44–1.71	1.36	1.25–1.49	1.30	1.23–1.39	1.14	1.07-1.22
Treatment with lipid-modifying agents	1.21	1.14–1.27	1.09	1.03–1.15	1.15	1.10–1.21	1.04	1.00–1.09
Drug-treated hypertension	1.27	1.21–1.34	1.13	1.07–1.19	1.22	1.17–1.28	1.08	1.03–1.13
Chronic Obstructive Pulmonary Disease (COPD)	1.49	1.42–1.57	1.46	1.39–1.54	1.59	1.52–1.67	1.54	1.47–1.61
Treated bronchial asthma	1.54	1.43–1.66	1.55	1.44–1.68	1.61	1.48–1.74	1.58	1.46–1.71
Liver diseases including chronic viral hepatitis	1.10	1.03–1.18	1.06	1.00–1.14	1.05	1.00–1.11	1.04	0.98–1.10
Treated diabetes	1.16	1.08–1.25	1.08	1.00–1.16	1.07	1.02–1.14	1.00	0.95–1.06
Treatment with immunosuppressive drugs	1.44	1.34–1.55	1.41	1.31–1.52	1.50	1.40–1.61	1.45	1.35–1.55
Hemiplegia	1.40	1.24–1.57	1.34	1.19–1.51	1.17	1.06–1.29	1.12	1.02–1.23
At least two of the above[2]	1.61	1.54-1.70	1.50	1.42-1.57	1.47	1.41-1.54	1.34	1.28-1.40
Indicators of unhealthy lifestyle								
Alcohol abuse	1.04	0.96-1.13	1.09	1.00–1.18	1.06	1.00–1.12	1.16	1.09–1.22
Smoking[1]	0.96	0.92–1.01	1.03	0.98–1.08	1.03	0.99–1.07	1.11	1.07–1.16
Obesity	1.15	1.09–1.22	1.12	1.06–1.19	1.01	0.97–1.06	0.98	0.93–1.03

[1] Information on smoking is obtained from the claims data via information on mental and behavioral disorders due to use of tobacco (ICD-10 GM: F17), tobacco or nicotine poisoning (T65.2) or by prescribing medication to treat nicotine dependence (ATC-Code: N07BA). As information on smoking is not routinely collected, the proportion of smokers is likely underestimated in German health claims data.

OR: Odds ratio, CI: Confidence interval.

[2] Less severe comorbidities (treatment with lipid-modifying agents and drug-treated hypertension) were not considered here.
